# Nutrition-Related Knowledge Graph Neural Network for Food Recommendation

**DOI:** 10.3390/foods13132144

**Published:** 2024-07-05

**Authors:** Wenming Ma, Mingqi Li, Jian Dai, Jianguo Ding, Zihao Chu, Hao Chen

**Affiliations:** 1 School of Computer and Control Engineering, Yantai University, Yantai 264005, China; lmq135831@s.ytu.edu.cn (M.L.); czh1126940012@s.ytu.edu.cn (Z.C.); 202200358067@s.ytu.edu.cn (H.C.); 2 Xinjiang Academy of Agricultural Sciences, Urumqi 830091, China; daij@xaas.ac.cn; 3 Institute of Agricultural Economics and Information, Xinjiang Academy of Agricultural Sciences, Urumqi 830091, China; djg@xaas.ac.cn

**Keywords:** food, heterogeneous graph, graph neural networks, recommendation systems

## Abstract

Food recommendation systems are becoming increasingly vital in modern society, given the fast-paced lifestyle and diverse dietary habits. Existing research and implemented solutions often rely on user preferences and past behaviors for recommendations, which poses significant issues. Firstly, this approach inadequately considers the nutritional content of foods, potentially leading to recommendations that are overly homogeneous and lacking in diversity. Secondly, it may result in repetitive suggestions of the same types of foods, thereby encouraging users to develop unhealthy dietary habits that could adversely affect their overall health. To address this issue, we introduce a novel nutrition-related knowledge graph (NRKG) method based on graph convolutional networks (GCNs). This method not only enhances users’ ability to select appropriate foods but also encourages the development of healthy eating habits, thereby contributing to overall public health. The NRKG method comprises two key components: user nutrition-related food preferences and recipe nutrition components. The first component gathers nutritional information from recipes that users show interest in and synthesizes these data for user reference. The second component connects recipes with similar nutritional profiles, forming a complex heterogeneous graph structure. By learning from this graph, the NRKG method integrates user preferences with nutritional data, resulting in more accurate and personalized food recommendations. We evaluated the NRKG method against six baseline methods using real-world food datasets. In the 100% dataset, the five metrics exceeded the performance of the best baseline method by 2.8%, 5.9%, 1.5%, 9.7%, and 6.0%, respectively. The results indicate that our NRKG method significantly outperforms the baseline methods, including FeaStNet, DeepGCN, GraphSAGE, GAT, UniMP, and GATv2, demonstrating its superiority and effectiveness in promoting healthier and more diverse eating habits. Unlike these baseline methods, which primarily focus on hierarchical information propagation, our NRKG method offers a more comprehensive approach by integrating the nutritional information of recipes with user preferences.

## 1. Introduction

Food recommendation systems [1,2,3] have traditionally been based on individual tastes, often lacking diversity. This can lead to unhealthy eating habits [4,5,6], as an over-reliance on a narrow range of foods can result in nutritional imbalances and deficiencies in essential vitamins and minerals. Consequently, to ensure comprehensive nutrient intake and maintain a healthy diet, it is crucial for food recommendation systems to promote dietary diversity, encouraging users to try a wide variety of foods. A diverse diet is essential for providing the necessary nutrients to maintain overall health.

In parallel with these dietary concerns, the rapid development and widespread adoption of information technology have increased the demand for personalized recommendations. Traditional food recommendation methods, which often rely on manual expertise or simple rule-based approaches, fail to meet the diverse needs of modern users. Therefore, leveraging technologies such as machine learning [7,8,9] and data mining [10,11,12] to construct personalized food recommendation models based on user preferences, historical behaviors, and food attributes has become a prevalent trend.

Based on the above, food recommendation systems play a crucial role in enhancing user experience by delivering personalized suggestions tailored to individual preferences. However, the current emphasis on personalization raises concerns regarding the potential impact on food diversity and the promotion of healthy eating habits. An excessive reliance on user historical data may inadvertently limit the variety of recommended foods and, in some cases, encourage unhealthy dietary choices. This study alleviates the critical gap in the existing literature by exploring how to balance the benefits of personalized food recommendations with the promotion of diverse and healthy dietary practices. We investigate whether integrating many nutrients into personalized recommendation methods can effectively mitigate these challenges. By doing so, this research aims to contribute novel insights into optimizing food recommendation systems to not only enhance user satisfaction but also foster healthier eating behaviors.

To mitigate these challenges, we propose a novel nutrition-related knowledge graph food recommendation method based on graph convolutional networks (GCNs). Specifically, we extracted seven key nutrients from recipe information—calories, total fat, saturated fat, sodium, protein, sugar, and carbohydrates—and standardized these to derive recipe nutritional components. Subsequently, we calculated the similarity between recipes based on their nutrient profiles, linking similar recipes to form a nutrition-related food similarity graph. By analyzing user–recipe interaction data, we identified recipes that users rated highly and calculated the average nutrient values of these recipes to establish user nutrition-related food preferences. This process culminated in the construction of a heterogeneous undirected graph comprising users, recipes, recipe nutritional components, user recipe ratings, and the nutrition-related food similarity graph. By applying attention mechanisms to recipes and their nutritional components, and integrating user preferences, we derived embeddings for both recipes and users. Ultimately, food recommendations were generated by learning through GCNs within the heterogeneous graph [13,14,15], combining user and recipe embeddings.

The contributions of our research are as follows:

- First, we introduce the concept of “recipe nutritional components,” enabling a systematic analysis of the nutritional value of recipes. This allows us to provide users with more accurate and comprehensive nutritional information, helping them better understand the nutritional content of their food and manage their dietary health. Additionally, this feature offers more options for users with special dietary needs or restrictions. Details are shown in Table 1;
- Second, we introduce the concept of “user nutrition-related food preferences.” By aggregating each user’s average nutritional preferences, we can tailor recipe recommendations to help them achieve their health goals. These personalized recommendations can be adjusted based on the user’s health status, goals, taste preferences, and dietary habits, providing recipe suggestions that better meet their needs and enhancing their satisfaction and loyalty to the recommendation system. Details are shown in Table 2;
- Third, we propose a novel nutrition-related knowledge graph food recommendation method based on a GCN. This method integrates recipe nutritional components and user nutrition-related food preferences to construct a complex heterogeneous graph model, capturing richer and more comprehensive information. Compared to traditional methods, our approach improves the efficiency and accuracy of recommendations, offering more personalized and precise food recommendation services. Details are shown in Table 3;
- Finally, we evaluated our method using a real-world food recommendation dataset (formerly GeniusKitchen). The experimental results clearly demonstrate that our method outperformed six baseline methods, highlighting its superior performance and potential. Details are shown in Table 4.

## 2. Related Work

This section reviews three key areas relevant to our work. First, we discuss collaborative filtering and content-based recommendation methods, which are foundational techniques in recommendation systems. Collaborative filtering leverages user behavior and interaction data to make personalized recommendations, while content-based methods use item features and descriptions to match user preferences. Second, we examine the application of graph neural networks in recommendation systems. These advanced models can capture and represent the intricate relationships between users and items, leading to more accurate and nuanced recommendations. Finally, we explore food-based recommendation methods, which tailor recommendations based on user tastes, food attributes, and nutritional needs, making them particularly effective for scenarios involving dietary preferences and personalized nutrition.

### 2.1. Collaborative Filtering and Content-Based Recommendations

Collaborative filtering and content-based recommendation methods are widely used strategies in recommendation systems. They generate personalized recommendations [16] by analyzing user behaviors, preferences, and item features.

Collaborative filtering relies on user behavior data and item data to suggest items that may interest users. It can be categorized into two types: item-based [17] and user-based [18]. Item-based collaborative filtering makes recommendations by comparing the characteristics and relationships among items, while user-based collaborative filtering does so by comparing the preferences and behaviors of users.

In contrast, content-based recommendation methods [19] suggest items similar to those users have previously liked by analyzing the attributes and features of items and user preferences. These methods primarily use item attributes such as tags, keywords, and descriptions, combined with users’ historical behavior data, to recommend items that align with their interests.

For instance, He et al. [20] introduced a recommendation system framework called Neural Collaborative Filtering (NCF), which leverages neural networks to learn from data. Unlike traditional collaborative filtering that uses inner products, NCF uses neural networks to learn any function, thereby enhancing the expressiveness and generalization of matrix factorization. To boost the non-linearity of NCF, they proposed using a multi-layer perceptron to learn the interactions between users and items.

Moreover, Wang et al. [21] proposed a novel method called Neural Graph Collaborative Filtering (NGCF). This approach propagates embeddings on graph data using the graph structure of users and items, effectively integrating collaborative signals into the entire embedding process. It also captures high-order connections in the user-item graph structure, addressing a significant limitation of traditional methods that do not adequately consider collaborative signals during the embedding process.

### 2.2. Graph Neural Network-Based Recommendations

Graph neural networks (GNNs) have emerged as a powerful tool for recommendation systems due to their ability to model complex relationships between users and items. GNNs leverage graph structures to propagate information and learn embeddings that capture the intricate interactions within the data.

For example, Zhang et al. [22] introduced a POI recommendation method known as the Hybrid Structure Graph Attention Network (HS-GAT). This method preprocesses data from multiple sources and creates two diverse graph datasets using users, user attributes, user POIs, and POI attributes. After merging the diverse graph data using dual graph attention operations, it constructs homogeneous graphs for user-POI and POI relationships. Finally, through the graph attention network (GAT), it incorporates the user embedding matrix and POI embedding matrix into the homogeneous graph to learn information and obtain the ultimate user and POI embeddings.

Similarly, Yin et al. [23] proposed a Generalized Collaborative Filtering (GCF) framework and simultaneously utilized deep graph neural networks for predicting links on bipartite graphs through information propagation, addressing the sparsity issue in recommendation systems. GCF aims to map users and items into a low-dimensional vector space. This mapping embeds geometric relationships that reflect the dynamic preferences between users and items. They also validated that traditional matrix factorization methods such as SVD and SVD++ can be interpreted within the GCF framework using node embeddings from graph neural networks. Moreover, they integrated attention mechanisms to handle the challenge of varying input sizes for each node in bipartite graphs, thereby enhancing the system’s ability to predict user–item interactions in sparse data scenarios.

### 2.3. Food-Based Recommendations

Food-based recommendation methods are specifically designed to cater to users’ tastes, food attributes, and nutritional needs. These methods are particularly suitable for scenarios related to food and dietary preferences, offering personalized recommendations that promote healthier eating habits.

For example, Ge et al. [24] introduced a new food recommender system that offers personalized recipe suggestions based on users’ ratings and tags. Their algorithm introduces tag-based matrix factorization and they designed a new HCI interaction applied to a mobile platform for recommending food recipes, which provides insights into preferred ingredients or the characteristics of foods favored by users. Their research highlights the significant role of tags in recommendation algorithms, which not only aid in modeling users and meals but also underscore how the judicious allocation and management of tags can be a crucial factor for the success of recommendation systems.

Chen et al. [25] proposed a novel method for recommending healthy foods that take into account users’ personalized health needs and dietary preferences. Initially, they constructed a collaborative recipe knowledge graph, which includes user–recipe interactions, recipe–ingredient associations, and other food-related information. Next, they developed a mechanism to evaluate the consistency between recipes and users’ health preferences, assessing the health alignment of recipes with users’ dietary preferences. Finally, based on these two components, they developed a health-aware food recommendation model using knowledge graph embeddings and multi-task learning techniques. This model employs a graph convolutional network to capture the semantic relationships between users and recipes in the collaborative knowledge graph. By integrating the losses from these two learning tasks, the model effectively learns user requirements in terms of both preferences and health, thereby improving the accuracy of recommendations.

Additionally, Gao et al. [26] argue that the complex relationships among ingredients, recipes, and users are crucial for recommendation systems. Therefore, they developed a method called FGCN that achieves precise recommendations through three types of information propagation: between ingredients, between ingredients and recipes, and between recipes and users. The information propagation mechanism adopted by the model extensively leverages the interactions among ingredients, recipes, and users. It employs multiple embedding propagation layers to model high-order connections and enhances representation learning, providing rich representations and more accurate recommendations.

Furthermore, Song et al. [27] proposed a self-supervised calorie-aware heterogeneous graph network recommendation method (SCHGN) that enhances the relationships between ingredients by considering the calorie content of food. This method constructs a heterogeneous directed graph representing the complex relationships among users, recipes, ingredients, and calories to clearly present these relationships. Through self-supervised ingredient prediction, the method explores the co-occurrence of ingredients in different recipes. Using hierarchical message passing, SCHGN learns calorie-aware user representations and calculates comprehensive user-guided recipe representations through an attention mechanism, thereby effectively capturing users’ preferences for the calorie content in food and providing more accurate recommendations.

Lastly, Rostami et al. [28] developed a hybrid food recommendation system that overcomes the limitations and shortcomings of previous food recommendation systems, such as ignoring ingredients, time constraints, new users, new foods, and community elements. In the first phase, the system uses graph clustering methods, and in the second phase, deep learning methods are employed to cluster users and foods, thereby achieving accurate grouping. Additionally, they adopted a comprehensive approach to address issues related to time and user communities. Based on these operations, the system significantly improved the quality of recommendations, helping users to adjust their eating habits and achieve healthier diets.

### 2.4. Summary

Table 5 and Table 6 provide a comprehensive comparison of our proposed work with several related works in the field. As illustrated, our method leverages a combination of a graph convolutional network (GCN) and graph attention network (GAT), which have not been extensively applied in previous works. While most related works use simpler machine learning techniques or one neural network, our work stands out by integrating multiple neural network models that incorporate both user preferences and a wide range of nutritional components. Unlike previous studies that often overlook user dietary preferences and nutritional details, our method provides a holistic approach by considering user nutrition-related food preferences and detailed nutritional components such as calories, total fat, saturated fat, sodium, protein, sugar, and carbohydrates. Additionally, our approach utilizes a heterogeneous undirected graph structure, which enhances the capability of our model to capture complex relationships within the data. This comprehensive and nuanced methodology significantly improves the accuracy and relevance of the recommendations provided by our system, offering a distinct advantage over existing methods.

In summary, the advancements in collaborative filtering, GNN-based methods, and specialized food-based recommendation approaches form the foundation of our research, guiding the development of a more accurate, diverse, and health-conscious food recommendation system.

## 3. Preliminary Knowledge and Problem Definition

In this section, we introduce the problem definition of our research and provide the necessary background knowledge to understand our study better. We have listed all the essential notations used in this study along with their definitions. They are presented in Table 7. The notations and the table itself were defined and compiled by us. Through Table 7, the meaning of each symbol can be clearly understood, which is crucial for comprehending the subsequent formulas and analyses in this study.

### 3.1. Problem Statement

To address the problem more clearly, we first summarize the drawbacks identified in the related works. This summary provides a comprehensive overview of the limitations present in existing research, emphasizing the need for our proposed approach. The main drawbacks are presented in Table 8.

By presenting this table, we aim to highlight the gaps in the current literature, thereby justifying the necessity of our research. The identified major drawbacks include the lack of consideration for various nutritional components and unhealthy recommendations due to an over-reliance on user preferences. These points underscore the areas where our study aims to contribute improvements. Our goal was to enhance recommendations by incorporating various nutritional components, building upon users’ personalized preferences to ensure not only greater precision but also improved healthiness.

### 3.2. Problem Definition

Our goal was to train a function F using the user–recipe interaction matrix Θ [29], a heterogeneous undirected graph G, recipe nutritional components ϑ, user nutrition-related food preferences P, a recipe information table Z, and additional parameters Π. This function aims to predict the likelihood of interaction between users and unfamiliar recipes, ultimately providing more personalized recommendation services. The function can be represented as follows:

(1) $${\hat{y}}_{uv}=F\left(u,r|\Theta ,G,P,\vartheta ,Z,\Pi \right),$$

where ${\hat{y}}_{uv}$ symbolizes the possibility of interaction, *u* stands for the user, and *r* denotes the recipe.

### 3.3. User–Recipe Interaction Matrix and Recipe Information Table

Consider a total of *M* users, denoted as $U=\{u_{1},u_{2},\ldots ,u_{M}\}$, and *N* recipes, denoted as $R=\{r_{1},r_{2},\ldots ,r_{N}\}$. The user–recipe interaction matrix Θ can be defined as:

(2) Θ={u,r|u∈U,r∈R},

where interactions between users and recipes are represented by (u,r). The actual label $y_{uv}$ indicates whether there is engagement between the user and the recipe $(y_{uv}=1$ for engagement; $y_{uv}=0$ for no interaction).

The recipe information table Z includes various details about recipes, such as descriptions, ingredients, food quantities, nutritional components, and labels. In this research, we utilized nutritional components to construct the recipe nutritional components ϑ, user nutrition-related food preferences P, the nutrition-related food similarity graph ϰ, and the heterogeneous undirected graph G.

### 3.4. Graph Neural Network

Graph neural networks (GNNs) are a specialized type of artificial neural network designed to analyze graph data within machine learning models [30,31,32]. Graph data comprise nodes (vertices) and edges, with nodes representing entities and edges denoting relationships between them. GNNs aim to understand these connections to perform tasks such as link prediction, node classification, and graph classification. Unlike conventional neural networks [33,34,35], GNNs consider the structure of nodes, enabling them to better capture specific and widespread information when dealing with graph data.

GNNs typically have multiple layers, each updating node representations by gathering information from nearby nodes. This aggregation operation can be implemented through message passing or graph convolution, among other methods. The development of GNNs has wide applications in fields such as recommendation systems, bioinformatics [36], and social network analysis [37]. The update rules used in our research are as follows:

(3) $$x_{i}^{\prime }=W_{1}x_{i}+W_{2}\sum_{j\in N(i)}e_{j,i}\cdot x_{j}.$$

where $x_{i}^{\prime }$ represents the updated features of node *i*, $e_{j,i}$ denotes the weight of the edge connecting node *j* to node *i*, $x_{i}$ and $x_{j}$ are the feature vectors corresponding to node *i* and node *j*, respectively, $W_{1}$ and $W_{2}$ are the weight matrices to be learned, and N(i) represents the collection of neighboring nodes of node *i*.

## 4. Proposed Method

**Figure 1:**
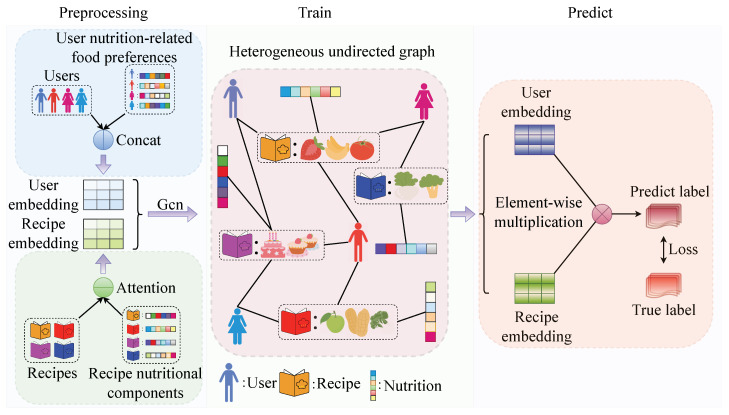
Overall architecture.

In this section, we provide a detailed explanation of our model, illustrated in Figure 1. We first present the general framework and design principles, including the data processing flow and model architecture. Then, we explain the graph construction process, detailing how nodes are connected and their main content. Following that, we discuss the process of information aggregation and update, explaining how to effectively integrate information from various nodes and update model parameters. Lastly, we introduce the optimization and algorithm section, describing the optimization methods and algorithmic processes we adopt for better training and tuning of our model. This lays the theoretical foundation for subsequent experiments and the result analysis.

### 4.1. Model Overview

**Figure 2:**
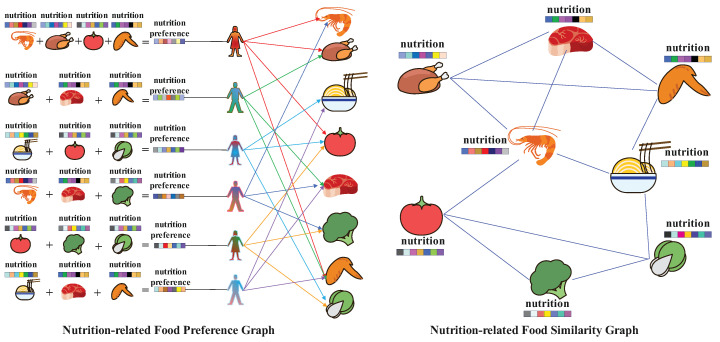
Nutrition-related user food knowledge graph.

During the heterogeneous undirected graph G construction phase, we obtain the nutritional components of each recipe from the recipe information table Z, including calories, total fat, saturated fat, sodium, protein, sugar, and carbohydrates, as the feature data θ of the recipe. Then, we standardize these features to transform them into a standard normal distribution with a mean of 0 and a standard deviation of 1, obtaining recipe nutritional components ϑ. Next, we use the similarity function to evaluate the analogousness between different recipes, thereby establishing a nutrition-related food similarity graph ϰ. Refer to Figure 2 for details. Finally, we construct heterogeneous undirected graph data G containing a user set U, a recipe set R, recipe nutritional components ϑ, user recipe ratings T, and a nutrition-related food similarity graph ϰ.

During the construction of the user nutrition-related food preferences P, we iterate through the user–recipe interaction matrix Θ; during this process, if a user *u* rates a recipe *r* as 5 points, we consider that recipe *r* as the user’s preferred recipe. If a user *u* has never rated a recipe with 5 points, we consider the first recipe they interacted with *r* (regardless of the rating) as their preferred recipe and only that recipe. After iterating through the entire user–recipe interaction matrix Θ, each user *u* will have a collection of preferred recipes *r*. Then, we calculate the average nutritional components for each preferred recipe *r*, obtaining the average nutritional components for each user’s preferred recipes as user nutrition-related food preferences P.

During the aggregation and update phase, we first apply an attention operation to the recipe vectors *r* and recipe nutritional components ϑ to obtain the final recipe embeddings $\tilde{r}$. We then use concatenation of user vectors *u* and user nutrition-related food preferences P to obtain the final user embedding $\tilde{u}$. We perform GCN operations on the recipe embeddings $\tilde{r}$ and the user embeddings $\tilde{u}$ in the heterogeneous undirected graph to learn information. The final prediction result is obtained by multiplying user embeddings $\tilde{u}$ and recipe embeddings $\tilde{r}$. Parameters are updated by comparing the prediction result with the actual result. This process is repeated until the algorithm converges.

### 4.2. Graph Construction

We first obtain all unique recipes based on the user–recipe interaction matrix Θ. Then, we extract the information of each recipe from the recipe information table Z. We take out seven types of nutrients for each recipe *r*, including calories, total fat, saturated fat, sodium, protein, sugar, and carbohydrates, forming a matrix of nutritional features for all recipes, which is the recipe’s feature data θ. We standardize the feature data matrix θ, transforming the data into a standard normal distribution with a mean of 0 and a standard deviation of 1:

(4) $$x_{std}=\frac{x-\mu }{\sigma },$$

where $x_{std}$ represents the standardized feature value, μ denotes the mean of the feature, σ stands for the standard deviation of the feature, and *x* represents the original feature value.

**Figure 3:**
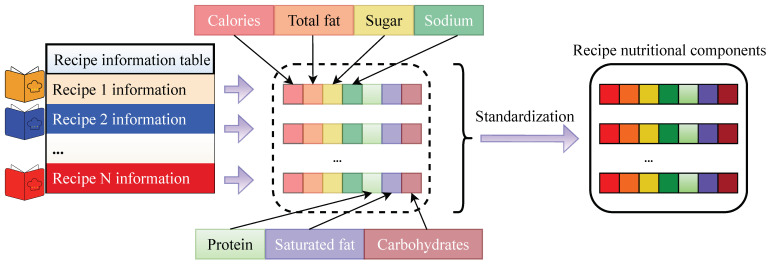
Recipe nutritional components.

The standardized feature data matrix, ϑ, represents the recipe nutritional components. The process is shown in Figure 3.

We use a similarity function to gauge the nutritional similarity between recipes:

(5) $$\cos=\frac{r_{A}\cdot r_{B}}{\left(\sqrt{r_{A}\cdot r_{A}}\right)\cdot \left(\sqrt{r_{B}\cdot r_{B}}\right)},$$

where $r_{A}$ and $r_{B}$ represent the nutrients of recipes A and B, respectively. We define a threshold of 0.98 to determine similarity. If the cosine similarity cos of two recipes exceeds 0.98, they are considered similar and added to the nutrition-related food similarity graph ϰ. After evaluating all recipes, we transpose the matrix to obtain the final nutrition-related food similarity graph ϰ, which contains all pairs of recipes with similar nutrients:

(6) $$\varkappa =\left[\begin{array}{c} \left[\begin{array}{ccccc} r_{1} & r_{2} & r_{3} & \ldots & r_{n} \end{array}\right],\\ \left[\begin{array}{ccccc} r_{5} & r_{7} & r_{8} & \ldots & r_{m} \end{array}\right] \end{array}\right],$$

where $r_{1}$ and $r_{5}$ represent a similar relationship.

We use the user set U, recipe set R, recipe nutritional components ϑ, user recipe ratings T, and nutrition-related food similarity graph ϰ to construct heterogeneous graph data. The final heterogeneous undirected graph data are G.

### 4.3. User Nutrition-Related Food Preferences

We traverse the user–recipe interaction matrix Θ. If a user *u* rates a recipe *r* with a score of 5, the recipe *r* is added to the user’s preferred recipe collection λ:

(7) $$\lambda_{u}=\left\{{r_{p}}_{1},{r_{p}}_{2},\ldots ,{r_{p}}_{n}\right\},$$

where $\lambda_{u}$ represents the preferred recipe collection for a specific user, and $r_{p}$ represents one of the user-preferred recipes.

For users who never rate a recipe as 5, the first recipe they interacted with *r* (regardless of the rating) is considered as their preferred recipe:

(8) $$\lambda_{u}=\left\{{r_{p}}_{1}\right\},$$

**Figure 4:**
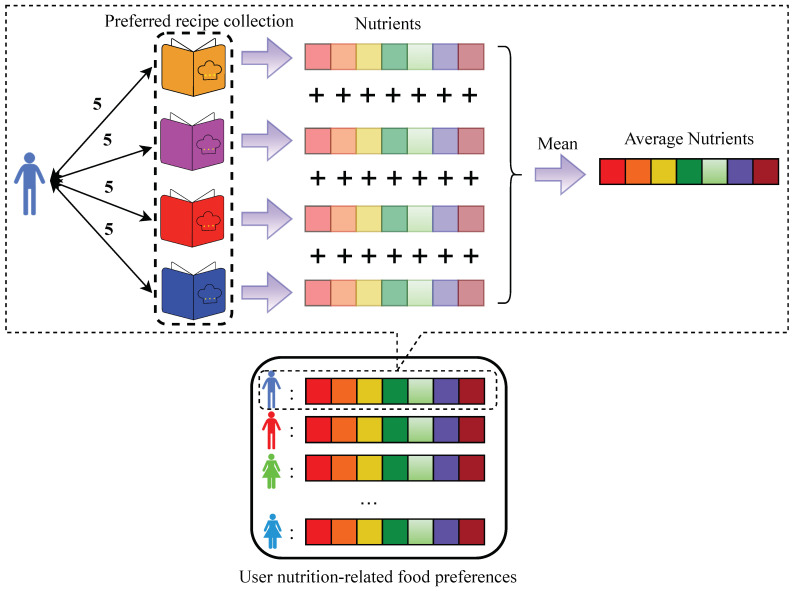
User nutrition-related food preferences.

After completing the traversal of the user–recipe interaction matrix Θ, all users will have a preferred recipe collection λ. We extract the seven nutrients corresponding to each recipe from the recipe information table Z and calculate the average value of each nutrient for all recipes in the preferred recipe collection λ, resulting in:

(9) $$P=\left[\begin{array}{c} u_{1}:\left[\begin{array}{ccccc} c_{1} & c_{2} & c_{3} & \ldots & c_{7} \end{array}\right],\\ u_{2}:\left[\begin{array}{ccccc} c_{1} & c_{2} & c_{3} & \ldots & c_{7} \end{array}\right],\\ \ldots \\ u_{n}:\left[\begin{array}{ccccc} c_{1} & c_{2} & c_{3} & \ldots & c_{7} \end{array}\right] \end{array}\right],$$

where *c* represents one of the seven nutrients after averaging operations. This gives us the user nutrition-related food preferences P, representing the nutrients preferred by each user based on their preferred recipes (Figure 4).

### 4.4. Aggregation and Update

We first use the function *f* to merge the recipe nutritional components ϑ with the recipe vector *r* to obtain a new recipe vector $r_{\vartheta }$:

(10) $$r_{\vartheta }=f\left(r,\vartheta \right),$$

where the function *f* denotes element-wise multiplication.

Next, we concatenate $r_{\vartheta }$ with the recipe vector *r* to obtain $r_{\vartheta }^{r}$:

(11) $$r_{\vartheta }^{r}=\mathrm{concat}\left(r_{\vartheta },r\right),$$

We then feature concatenate $r_{\vartheta }$ with recipe nutritional components ϑ to obtain $r_{\vartheta }^{\vartheta }$.

(12) $$r_{\vartheta }^{\vartheta }=\mathrm{concat}\left(r_{\vartheta },\vartheta \right),$$

We then element-wise add $r_{\vartheta }^{r}$ and $r_{\vartheta }^{\vartheta }$ to obtain γ:

(13) $$\gamma =\left(r_{\vartheta }^{r}+r_{\vartheta }^{\vartheta }\right),$$

We perform attention operations on $r_{\vartheta }^{r}$, $r_{\vartheta }^{\vartheta }$, and γ to obtain the recipe embedding $\tilde{r}$:

(14) $$\tilde{r}=\mathrm{MA}\left(r_{\vartheta }^{r},r_{\vartheta }^{\vartheta },\gamma \right),$$

where **MA** is a multi-head attention mechanism. Its calculation formula is as follows:

(15) $$\mathrm{MA}\left(Q,K,V\right)=\mathrm{concat}\left(head_{1},head_{2},\ldots ,head_{h}\right)W^{O},$$

(16) $$head_{i}=\mathrm{Attention}\left(Q_{i},K_{i},V_{i}\right),$$

(17) $$\mathrm{Attention}\left(Q,K,V\right)=\mathrm{softmax}\left(QK^{T}/\sqrt{d_{k}}\right)V,$$

where $W^{O}$ represents the output weight matrix, the input query matrix is *Q*, the key matrix is *K*, and the value matrix is *V*. They are transformed linearly to obtain $Q^{\prime }=QW^{Q}$, $K^{\prime }=KW^{K}$, and $V^{\prime }=VW^{V}$, where $W^{Q}$, $W^{K}$, and $W^{V}$ are weight matrices. Then, $Q^{\prime }$, $K^{\prime }$, and $V^{\prime }$ are respectively split into *h* heads, namely, $Q_{1},Q_{2},\ldots ,Q_{h}$, $K_{1},K_{2},\ldots ,K_{h}$, $V_{1},V_{2},\ldots ,V_{h}$, where each head has a dimension of:

(18) $$d_{k}=d_{model}/h.$$

For each group of users *u* entering the model, we first traverse the total user nutrition-related food preferences P to query the nutritional components *p* preferred by each user. Then, we combine these preferred recipe nutritional components into the user nutrition-related food preferences batch $P_{s}$ for this batch of users.

(19) $$P_{s}=\left[\begin{array}{c} p_{1},\\ p_{2},\\ \ldots \\ p_{n} \end{array}\right],$$

(20) $$p_{i}=\left\{c_{1}\;c_{2}\;c_{3}\;\ldots \;c_{7}\right\},$$

where $c_{i}$ represents the mean value of a certain nutrient component.

Then, the user vector *u* is concatenated with the user nutrition-related food preferences batch $P_{s}$ to obtain the user embedding $\tilde{u}$.

(21) $$\tilde{u}=\mathrm{concat}\left(u,P_{s}\right),$$

The final adoption of the GraphConv layer is utilized to learn and update the features of the user embedding $\tilde{u}$ and recipe embedding $\tilde{r}$ on the heterogeneous undirected graph G, predicting the relevance by combining the features of users and recipes. The final predicted result $S_{\tilde{r}}^{\tilde{u}}$ is obtained by putting the recipe embedding $\tilde{r}$ and user embedding $\tilde{u}$ into the function *f*.

(22) $$S_{\tilde{r}}^{\tilde{u}}=f\left(\tilde{u},\tilde{r}\right),$$

Then, updating the parameters by comparing the predicted label with the true label. The above process is repeated until the algorithm converges.

### 4.5. Optimization and Algorithm

The final prediction label $\hat{y}$ is obtained by compressing $S_{\tilde{r}}^{\tilde{u}}$ to the range of 0–1 using the sigmoid function σ.

(23) $$\hat{y}=\sigma \left(S_{\tilde{r}}^{\tilde{u}}\right)=\frac{1}{1+e^{-S_{\tilde{r}}^{\tilde{u}}}},$$

The comprehensive loss function is:

(24) $$L\left(y,\hat{y}\right)=-\frac{1}{N}\sum_{i=1}^{N}\left[y_{i}\cdot \log\left({\hat{y}}_{i}\right)+\left(1-y_{i}\right)\cdot \log\left(1-{\hat{y}}_{i}\right)\right].$$

where *N* represents the sample size, $y_{i}$ is the actual label of the *i* th sample, ${\hat{y}}_{i}$ is the predicted label of the *i* th sample, and log denotes the natural logarithm.

The primary steps of the algorithm are outlined below: First, a function named “Obtain-user-preference-nutritional-components” is defined, and its role is to obtain the user nutrition-related food preferences $P_{s}$ for the group of users based on the entire user nutrition-related food preferences P.

During the iteration process of the NRKG algorithm, for each user–recipe interaction pair (u,r), the following operations are carried out:

- The “Obtain-user-preference-nutritional-components” function is called to obtain the user nutrition-related food preferences $P_{s}$;
- The attention mechanism function Atten is used to weight the recipe *r* and its nutritional components ϑ to obtain the recipe embedding $\tilde{r}$;
- The user *u* and user nutrition-related food preferences $P_{s}$ are concatenated to obtain the user embedding $\tilde{u}$;
- The graph convolution network function Gcn operates on the heterogeneous undirected graph G to perform graph convolution on $\tilde{u}$ and $\tilde{r}$, resulting in new representations $\tilde{u}$ and $\tilde{r}$;
- The function *f* is then used to compute the predicted label ${\hat{y}}_{uv}$.

Ultimately, the algorithm parameters are adjusted using the gradient descent optimization method. When the NRKG algorithm converges, it returns the prediction function F. Details are shown in Algorithm 1.
**Algorithm 1:** NRKG      **Input**: User–recipe interaction matrix Θ; heterogeneous undirected graph G;                 recipe nutritional components ϑ; user nutrition-related food preferences P;                  recipe information table Z; additional parameters Π;
                 training parameters: ${\left\{W_{i},b_{i}\right\}}_{i=1}^{H}$,${\left\{u\right\}}_{u\in U}$, ϕ, ${\left\{r\right\}}_{r\in R}$;                 hyperparameters: Gcn(·),f(·),Atten(·), concat(·)      **Output**: Prediction function F(u,r|Θ,G,P,ϑ,Z,Π) 
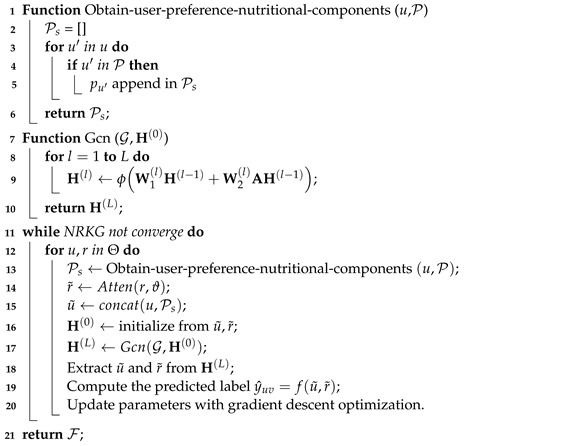


## 5. Experiments

This section details the experimental setup, including the environment configuration, dataset selection, and evaluation metrics. We also compare our method against various baselines and present the results. Furthermore, we delve into the hyperparameter analysis and conduct ablation studies to demonstrate the effectiveness of our approach.

### 5.1. Experimental Settings and Datasets

For our experimental setup, we relied on a robust stack of tools and libraries, ensuring a stable and reproducible environment. We utilized the PyTorch framework, a popular choice for deep learning tasks, alongside Python 3.9.16 for scripting and Torch 1.13.1 + cu116 for GPU acceleration. Additionally, we leveraged PyG 2.5.3 for graph neural network operations, Pandas 1.5.3 for data manipulation, and NumPy 1.23.5 for numerical computations. By employing this suite of tools, we ensured compatibility and efficiency throughout the experimentation process.

To assess the efficacy of our proposed method, we turned to real-world datasets, which offer diverse and complex scenarios for evaluation. One such dataset is the **Food Recommender** dataset (https://www.kaggle.com/code/aayushmishra1512/food-recommender accessed on 5 May 2024) [38], sourced from Food.com (formerly GeniusKitchen). This comprehensive dataset comprises an extensive collection of over one hundred and eighty thousand recipes and seven hundred thousand recipe reviews spanning eighteen years. With its rich repository of user engagements and recipe submissions, it serves as an ideal testbed for recommendation system research.

In our experiments, we focused on a carefully selected subset of the Food Recommender dataset, consisting of 10% of the interaction data. This subset encompasses 41,087 users, 63,544 recipes, and 113,236 interactions, providing a representative sample of user behavior and preferences. To ensure a robust evaluation, we employed a standard split strategy, dividing the dataset into training, validation, and test sets in a 6:2:2 ratio. This partitioning scheme allowed us to train our model on a sizable portion of the data while retaining separate subsets for validation and final evaluation, thus ensuring an unbiased performance assessment.

### 5.2. Baselines

For comparison with our proposed method, we considered six baseline approaches: FeaStNet, GraphSAGE, GAT, UniMP, DeeperGCN, and GATV2, each offering unique strategies for recommendation tasks:

- **FeaStNet** [39] introduces a dynamic graph-convolution operator, which dynamically creates associations between filter weights and graph neighborhoods using learned features. This approach contrasts with traditional static coordinate-based methods, offering a novel perspective on graph-based recommendation;
- **GraphSAGE** [40] leverages node feature data efficiently by embedding individual nodes and aggregating neighborhood features, enabling the generation of node embeddings for new nodes with enhanced efficiency;
- **GAT** [41] utilizes masked self-attention layers to address limitations of prior methods in handling graph-structured data. By allowing nodes to attend to neighborhood features and assign varying weights without costly matrix operations, GAT achieves expressive modeling of graph structures;
- **UniMP** [42] employs holistic message passing and a masking label prediction strategy to prevent overfitting. By integrating label propagation and local features using Graph Transformer networks, UniMP provides a robust approach for training and inference in recommendation systems;
- **DeeperGCN** [43] introduces differentiable generalized aggregation functions and employs normalization layers and pre-activation residual connections. This facilitates effective training of deep graph convolutional networks, enhancing their performance in recommendation tasks;
- **GATV2** [44] introduces dynamic graph attention to overcome the limitations of static attention in GAT. By enabling more expressive modeling of graph structures, GATV2 enhances the adaptability and performance of graph-based recommendation systems.

These baseline methods provided a diverse set of approaches for comparison with our proposed model, enabling a comprehensive evaluation of recommendation performance across different methodologies.

### 5.3. Evaluation Metrics

In simpler terms, AUC is a metric that assesses how well a binary classification model can distinguish between positive and negative classes. The closer the AUC value is to 1, the better the model performance; conversely, a lower value indicates poorer model performance. The formula can be expressed as:

(25) $$\mathrm{AUC}=\frac{1}{2}\sum_{i=1}^{k-1}\left({\mathrm{TPR}}_{i}+{\mathrm{TPR}}_{i+1}\right)\cdot \left({\mathrm{FPR}}_{i+1}-{\mathrm{FPR}}_{i}\right),$$

The total number of positive and negative samples is denoted by *k*. The true positive rate of the *i*-th sample, sorted by the predicted probability, is denoted as FPR, and the false positive rate is denoted as TPR.

Precision evaluates the accuracy of positive instances, indicating the ratio of true positive instances to all positive instances. The specific calculation is as follows:

(26) $$\mathrm{Precision}=\frac{\mathrm{True}\;\mathrm{Positives}}{\mathrm{True}\;\mathrm{Positives}+\mathrm{False}\;\mathrm{Positives}},$$

Accuracy assesses the overall model effectiveness in accurately forecasting both positive and negative classes, indicating the proportion of correct predictions. The specific calculation is as follows:

(27) $$\mathrm{Accuracy}=\frac{\mathrm{True}\;\mathrm{Positives}+\mathrm{True}\;\mathrm{Negatives}}{\mathrm{Total}\;\mathrm{Predictions}},$$

Recall is a metric that assesses whether a model can correctly identify all positive instances. It demonstrates the proportion of true positive instances in all positive instances after being processed by the model. The specific calculation is as follows:

(28) $$\mathrm{Recall}=\frac{\mathrm{True}\;\mathrm{Positives}}{\mathrm{True}\;\mathrm{Positives}+\mathrm{False}\;\mathrm{Negatives}},$$

F1 is a single metric, but it combines two other metrics: precision and recall. It evaluates the overall performance of a model by combining precision and recall. The specific calculation is as follows:

(29) $$F1=2\times \frac{\mathrm{Precision}\times \mathrm{Recall}}{\mathrm{Precision}+\mathrm{Recall}}.$$

### 5.4. Comparative Experiment

Through Table 9 and Table 10, we observe that FeaStNet, DeeperGCN, GraphSAGE, GAT, UniMP, GATV2, and our proposed method exhibited varying degrees of performance across different proportions of the Food dataset.

Our method demonstrated a slight disadvantage in the recall and F1 metrics compared to the FeaStNet method on the 10% dataset. Additionally, our method’s recall metric (0.6784) was marginally lower than that of the FeaStNet method (0.6800) on the 50% dataset. This discrepancy may stem from our proposed user nutrition-related food preferences module and recipe nutrition component module not fully leveraging their advantages due to insufficient information. The subsequent performance further validated this hypothesis. However, our proposed method showcased significant advantages in metrics such as AUC, accuracy (ACC), precision, recall, and F1 on the 80% and 100% datasets. Particularly in the 100% dataset, these metrics exhibited notable improvements of 2.8%, 5.9%, 1.5%, 9.7%, and 6.0%, respectively.

The exceptional performance of our method is primarily attributed to our proposed user nutrition-related food preferences module and recipe nutrition component module. These modules not only thoroughly explore user preferences for recipes but also accurately capture the nutritional characteristics of recipes, thereby enhancing the recommendation of recipes tailored to users’ tastes and nutritional requirements. Consequently, our method excelled across different proportions of the dataset and achieved optimal performance with 100% data, further confirming the effectiveness and reliability of our approach. In conclusion, our proposed method exhibited promising application prospects and superior performance in food recommendation systems.

### 5.5. Loss Function Analysis

We assessed our proposed method against several others by examining the changes in their training loss during the training process. By contrasting the training loss trends of different methods, we gained insights into their efficiency and generalization capabilities during optimization. Through visualizing the variations in training loss, we could clearly observe the performance of each method during training and extract valuable insights from the comparison.

**Figure 5:**
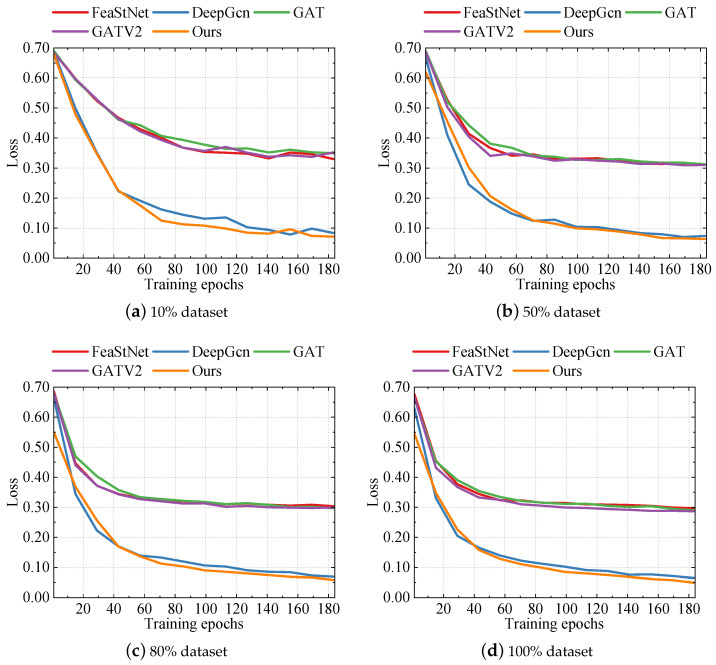
Training loss.

According to Figure 5, we can observe the training loss performance of five different methods within 183 epochs. From the figure, it is evident that our proposed method exhibited a lower training loss, whether on an 80% dataset or a 100% dataset in the initial stage, converged rapidly, and maintained a relatively stable downward trend. In comparison, our method showed a lower training loss in each epoch compared to other methods, reflecting its advantages concerning model convergence speed and efficiency. In the initial few epochs of the 100% dataset, our method reached a loss of 0.5456 in the first epoch, while GAT, DeepGcn, FeaStNet, and GATV2 reached losses of 0.6618, 0.6299, 0.6765, and 0.6667 respectively.

Furthermore, as the training progressed, our method was able to reach lower loss levels more quickly. By the 169th epoch, the loss of our method was 0.0580, while the losses of GAT, DeepGcn, FeaStNet, and GATV2 were 0.2949, 0.0715, 0.2993, and 0.2883, respectively. This indicates the effectiveness and robustness of our method in the model optimization process.

These results clearly illustrate the capability of our proposed method in the training process, laying a strong foundation for the further exploration and analysis of experimental results.

### 5.6. Ablation Experiment

We validate the effectiveness of our proposed module, assess the impact of each component on the model’s performance, and analyze and discuss the findings.

**Figure 6:**
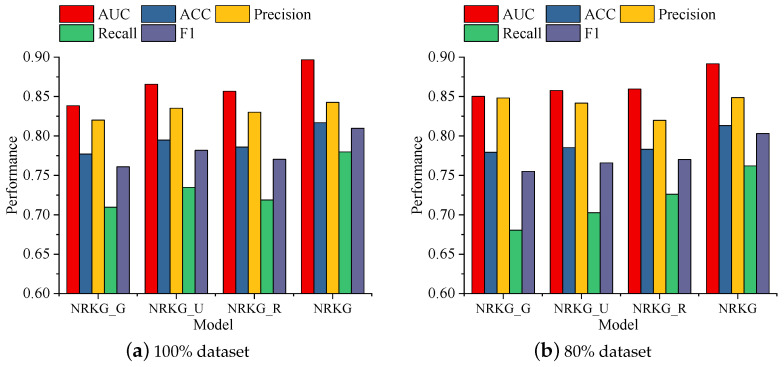
Different modules had different effects on NRKG.

According to Figure 6, we compared multiple variants of our proposed model to assess the effect of adding different modules on model performance. First was NRKG-G, which is the original algorithm without any additional modules. Then came NRKG-U, where we added a module for user nutrition-related food preferences. Next was NRKG-R, where we added a module for recipe nutritional components. Finally, there was NRKG, which incorporates both the user nutrition-related food preferences module and the recipe nutritional component module.

According to the performance on the 80% dataset and 100% datasets based on five metrics (AUC, ACC, precision, recall, and F1), NRKG-G exhibited the lowest performance across all metrics, indicating poorer performance when no additional information was input into the model. NRKG-U showed improvement over NRKG-G across all metrics, especially notable in AUC and F1, suggesting a positive impact of incorporating the user nutrition-related food preferences module on model performance. Similarly, NRKG-R demonstrated a significant improvement over NRKG-G, particularly in AUC and F1, indicating a positive impact of incorporating the recipe nutritional component module on model performance. The final NRKG method performed the best across all metrics, especially achieving high scores in terms of AUC and F1 scores on the 100% dataset with 0.8967 and 0.8098, and on the 80% dataset with 0.8916 and 0.8031, respectively, further validating the effectiveness of simultaneously incorporating both the user nutrition-related food preferences module and the recipe nutritional component module.

In summary, both the user nutrition-related food preferences module and the recipe nutritional component module were useful, and our proposed final NRKG method achieved significant performance improvements across multiple metrics, demonstrating its effectiveness and superiority.

### 5.7. Hyparameter Analysis

In this subsection, we evaluate and discuss the hyperparameters of the model by comparing the impact of hidden channel numbers, the impact of aggregate neighbor numbers, and the impact of the number of aggregation layers. By selecting and adjusting the hyperparameters and analyzing the research results, we optimized the performance and training process of the model, improving the model’s capacity for generalization and its speed of training. The work in this subsection helped to deepen our comprehension of the impact of model hyperparameters on model execution and the training process, providing important references for further optimizing the design and training of the model.

#### 5.7.1. Impact of the Number of Hidden Channels

**Figure 7:**
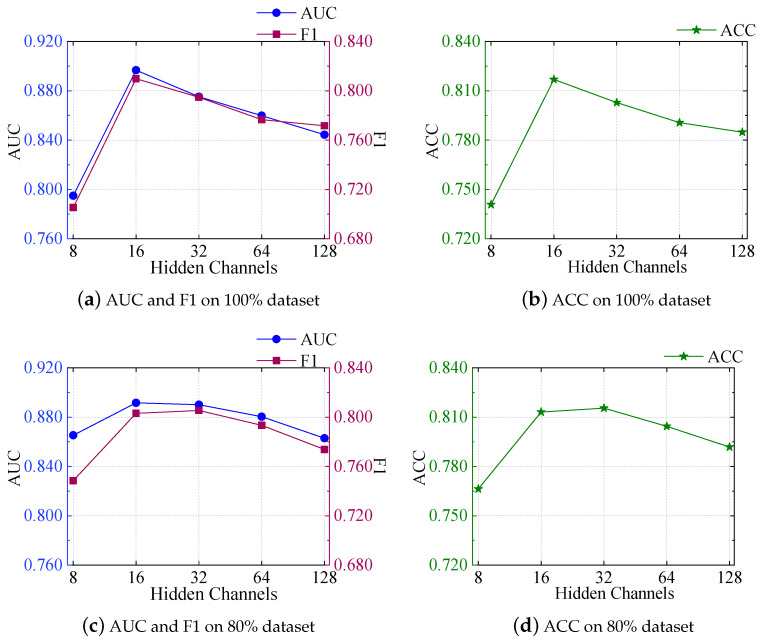
The impact of different hidden channel sizes on AUC, F1, and ACC metrics.

According to Figure 7, we can see that, as the number of hidden channels rose, both the 80% dataset and the 100% dataset models showed certain trends in AUC, F1, and ACC metrics. Initially, on the 100% dataset, with the increase in hidden channels from 8 to 16, the AUC significantly improved from 0.7949 to 0.8967, and the F1 score and ACC also exhibited a similar trend, increasing from 0.7052 to 0.8098 and from 0.7408 to 0.8169. However, further increases did not lead to additional performance gains. Although the AUC remained relatively high at 32 hidden channels (0.8752), it slightly decreased at 64 and 128 hidden channels to 0.8598 and 0.8444, respectively. Moreover, the F1 score slightly improved after increasing to 32 hidden channels but decreased slightly at 64 and 128 hidden channels to 0.7766 and 0.7716, respectively. The ACC also showed a downward trend, with values of 0.7906 and 0.7848, respectively.

On the 80% dataset, the same results were obtained. All metrics initially improved, with the AUC metric starting to decrease after the number of hidden channels reached 16, while the F1 and ACC metrics began to decline after the number of hidden channels reached 32.

This indicates that increasing the number of hidden channels can improve model performance to a certain extent, but excessive increases may cause performance to saturate or even decline, likely due to the model overfitting the data beyond a certain point. Therefore, when selecting the number of hidden channels, it is necessary to find an equilibrium between model intricacy and performance to fully utilize the model’s expressive capacity while avoiding overfitting.

#### 5.7.2. Impact of the Number of Neighbors

**Figure 8:**
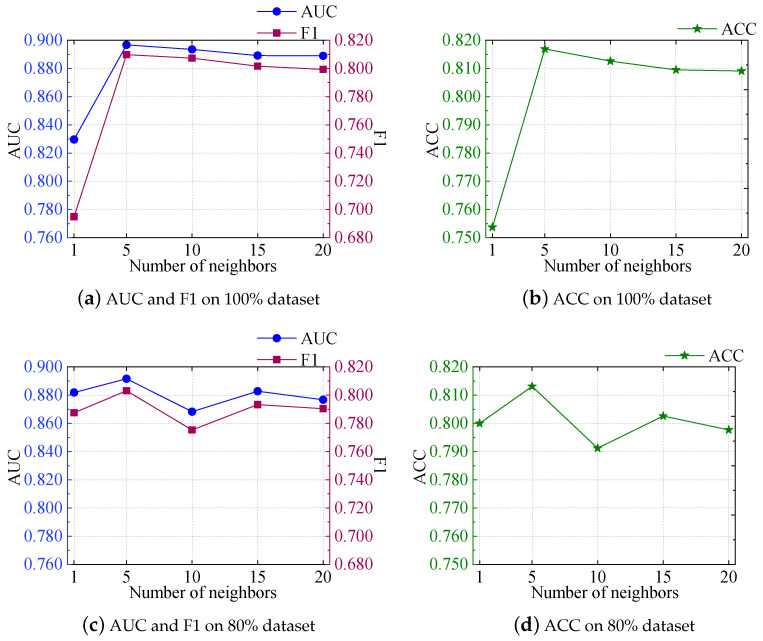
The impact of different neighbor numbers on AUC, F1, and ACC metrics.

According to Figure 8, it is evident that, as the number of neighbors increased on the 100% dataset, the model’s performance showed the propensity to first increase and then decline in the AUC, F1, and ACC metrics. Specifically, when the number of neighbors was five, the AUC, F1, and ACC metrics were 0.8967, 0.8098, and 0.8169, respectively, which were 8.1%, 16.5%, and 8.3% higher than when the number of neighbors was one. A notable performance boost can be observed, but as the quantity of neighbors continued to increase to 10, 15, and 20, the AUC, F1, and ACC metrics gradually decreased to 0.8889, 0.7993, and 0.8091, respectively.

On the 80% dataset, it was a different scene. All metrics reached their maximum values when the quantity of aggregating neighbors was set to five, and then decreased. There was a slight increase when the quantity of aggregating neighbors was set to 15, but the trend went down again when the quantity of aggregating neighbors was set to 20.

These trends indicate that, when choosing the appropriate number of neighbors, it is necessary to balance the model’s complexity and performance. When the model selects too few neighbors, it may lead to underfitting during training. However, when the model chooses too many neighbors, it may result in overfitting during training. Therefore, in practical applications, it is important to choose the most favorable number of neighbors to accomplish better performance.

#### 5.7.3. Impact of the Number of Aggregation Layers

**Figure 9:**
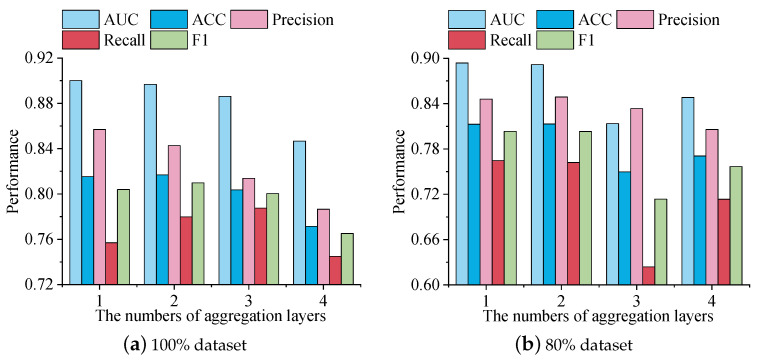
The impact of different numbers of aggregation layers on metrics.

According to Figure 9, we can see that, as the aggregation layers increased, the performance of the model showed different trends. First, on the 100% dataset, in terms of AUC, as the aggregation layers increased, the AUC value gradually decreased, indicating a weakening of the model’s predictive ability. At the same time, the ACC was higher when there were one and two aggregation layers, but showed a decreasing trend as the aggregation layers increased. However, precision and recall fluctuated at different aggregation layers. Precision and recall reached their highest values when there were two aggregation layers, but slightly decreased when there were three and four aggregation layers.

On 80% of the dataset, there were slight differences. The trends with one, two, and three layers were generally similar to the 100% dataset, but with four layers, the AUC, ACC, recall, and F1 metrics improved compared to three layers.

Considering the performance trends of various metrics, we can conclude that, when choosing the number of aggregation layers, it is necessary to balance the changes in various metrics to achieve a balanced performance. In the current dataset, when there were two aggregation layers, the model performed well concerning accuracy, precision, and recall, so it can be considered a suitable choice. However, it is significant to note that different datasets and projects may have an impact on the choice of aggregation layers, so further optimization and adjustment may be needed in practical applications.

## 6. Case Study

This section provides a detailed overview of two case studies aimed at further elucidating the effectiveness and superiority of our proposed method. Firstly, we demonstrate the visualization of embeddings and compare the aggregation of the original embeddings with those processed by our method. Secondly, we delve into the impact of incorporating user nutrition-related food preferences on the method’s accuracy. We present several user instances for comparison and conduct detailed analyses of them. Through these case studies, a clearer understanding of the advantages of our proposed method can be gained.

### 6.1. Visualization of Embedding

**Figure 10:**
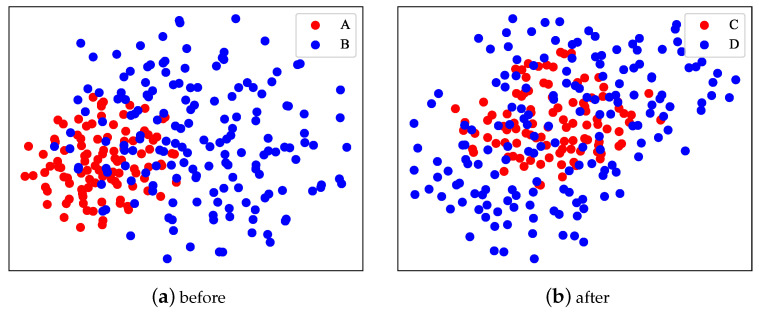
Visualization of embedding.

According to Figure 10, in our visualization results, we compared four different types of embeddings: *A* represents original user embeddings; *B* represents original recipe embeddings; *C* represents user embeddings concatenated with user nutrition-related food preferences P; and *D* represents recipe embeddings processed through an attention mechanism.

Upon observing the visualization results, we can see that the original user and recipe embeddings (*A* and *B*) were relatively scattered in the visualization space, indicating a weak or difficult-to-capture relationship between them. This may be because the original embeddings did not fully consider the correlation between users and recipes.

However, the processed embeddings (*C* and *D*) were closer in the visualization space, even showing clustering, which indicates that the processed embeddings more closely captured the relationship between users and recipes. This reflects the advantage of our method, which combines user-personalized preferences with recipe features, and utilizes an attention operation to better acquire the correlation between them, thereby boosting the performance and accuracy of the method.

In conclusion, by comparing the visualization results of different types of embeddings, we can clearly see that the processed embeddings better reflected the relationship between users and recipes, further validating the efficiency and advantage of our proposed method.

### 6.2. User Nutrition-Related Food Preferences Analysis

To demonstrate the contribution of user nutrition-related food preferences to our method, we selected several users from the test set to examine their user nutrition-related food preferences and the nutritional composition of recipes they interacted with, and we calculated the fit between the two sets of data. The method we employed involved computing the correlation coefficient between the two sets of data—the Pearson correlation coefficient. This indicator measures the strength of the relationship between two sets of data. Its values range from −1 to 1. When the value is closer to −1, it indicates a negative correlation between the two datasets. When the value is closer to 0, it suggests a weak correlation between the two datasets. A value closer to 1 indicates a positive correlation between the two datasets.

According to the information in Table 11, the first five users listed the nutritional components of the recipes they interacted with and their own user nutrition-related food preferences. The Pearson correlation coefficients ρ were all around 0.99, indicating a very strong correlation. For the user David, we listed a recipe he did not interact with, and we can see that the Pearson correlation coefficient ρ was 0.8101, which was lower compared to the previous users, indicating a poor correlation.

This reflects the contribution of the user nutrition-related food preferences proposed by us to our method. There was a close relationship between the recipe preferences of these users and the nutritional components they selected, which further validates the effectiveness and importance of the user nutrition-related food preferences introduced in our method.

## 7. Discussion

In this section, we comprehensively summarize our research, delving into the advantages and outcomes achieved, as well as the potential impacts. Moreover, we also emphasize some limitations within the research and propose directions for improvement. Through such enhancements, we gain a better understanding of the significance and constraints of the research, providing valuable references and insights for future research endeavors.

### 7.1. Advantages and Achievements

Our research combines recipes that users interacted with, reflecting food preferences while considering the seven essential nutrients found in all recipes—calories, total fat, saturated fat, sodium, protein, sugar, and carbohydrates. This research aimed to balance personalized food recommendations while promoting diverse and healthy dietary habits. We proposed the concept of “recipe nutritional components” and introduced the concept of “user nutrition-related food preferences”. Integrating these two concepts, we developed a novel food recommendation method based on a GCN for nutrition-related knowledge graphs. This method integrates recipe nutritional components with user-specific nutritional food preferences, constructing complex heterogeneous graph models to obtain richer and more comprehensive information. We validated the effectiveness of our method on the Food Recommender dataset through experiments. When compared with baseline models such as FeaStNet [39], DeepGcn [43], GraphSAGE [40], GAT [41], UniMP [42], and GATV2 [44], our NRKG model demonstrated significant advantages across four datasets with different proportions based on five metrics: AUC, ACC, precision, recall, and F1. Particularly on the 80% and 100% datasets, our model outperformed all baseline models in terms of performance. The advantage of our model can be attributed to the concepts we introduced: recipe nutritional components and user nutrition-related food preferences. By integrating nutritional components into recipes and highlighting users’ personalized food choices, our method leverages these factors synergistically. This demonstrates the accuracy of our recommendation method. Moreover, because all recipes incorporate a variety of nutrients, it also encourages people to choose more nutritious recipes, promoting healthier dietary habits.

Based on the above, our research yielded several notable advantages and achievements. We successfully developed a novel recommendation method that demonstrates remarkable efficacy in predicting and recommending personalized food choices. Through a meticulous analysis and interpretation of the results, we illustrated the potency and efficiency of our approach, particularly in enhancing recommendation accuracy. By integrating nutritional components into our recommendation system, we provided users with more targeted and beneficial dietary advice, contributing to the promotion of healthier eating habits. Furthermore, our findings underscore the potential benefits of incorporating food nutritional components into recommendation methods, thereby paving the way for advancements in personalized nutrition and dietary planning.

In practice, the approach we propose provides a more practical tool for food-related applications [45]. This tool not only helps users choose healthier dietary habits based on personalized tastes but also significantly enhances user comfort and reliance on the application.

### 7.2. Limitations and Constraints

Despite the successes of our research, certain limitations must be acknowledged. Firstly, our reliance on a single dataset may have introduced biases into our findings, limiting the generalizability of our results. Future research should aim to validate our method on datasets with larger scales and more diverse data to ensure robustness and reliability. Secondly, the limited scope of nutritional components [46] considered in our method may result in deviations in certain recommendations. Expanding the range of nutritional components considered could enhance the nutritional balance of recommended recipes, thereby improving the overall effectiveness of our method. Lastly, our recommendations do not currently account for users’ special circumstances, such as dietary restrictions or allergies. Incorporating these factors into our method would enable more tailored and personalized recommendations [47], thereby enhancing user satisfaction and engagement.

### 7.3. Directions for Improvement

To address the aforementioned limitations and further enhance the relevance and effectiveness of our research, several directions for improvement can be considered. Firstly, future studies should focus on validating our method on diverse datasets to ensure its robustness and generalizability across different contexts. Additionally, expanding the range of nutritional components considered in our method could improve the accuracy and relevance of recommendations, ultimately enhancing user satisfaction. Furthermore, incorporating users’ special circumstances, such as dietary restrictions [48] or allergies [49], into our recommendation system would enable more personalized and tailored recommendations, thereby increasing user engagement and adherence.

Through these proposed enhancements, we aim to further advance the effectiveness and reliability of our recommendation method, ultimately contributing to the advancement of personalized nutrition and dietary planning. By addressing these limitations and exploring new avenues for improvement, we can continue to make significant strides in the field of food recommendation systems and personalized nutrition guidance.

## 8. Conclusions

This study addresses the challenge of providing personalized healthy food recommendations by introducing the nutrition-related knowledge graph (NRKG) convolutional network method. The NRKG method incorporates two novel modules, namely, user nutrition-related food preferences and recipe nutritional components, within a GCN framework. Through extensive experimentation on the Food Recommender dataset, we compared the NRKG method with several baseline methods, including FeaStNet, GraphSAGE, GAT, UniMP, DeeperGCN, and GATV2. Our results consistently demonstrated the superior performance of the NRKG method across different dataset ratios.

The experimental findings highlight the NRKG method’s ability to offer more diverse food choices, thereby enhancing users’ dietary experiences and improving their health conditions. By accurately predicting user nutrient preferences, the NRKG method facilitates more personalized dietary recommendations, catering to individual needs and preferences. Moreover, this research introduces novel insights and methodologies for the advancement and implementation of food recommendation systems, holding practical significance and fostering further developments in the field.

We anticipate that this study will contribute positively to the design and enhancement of future dietary recommendation systems, fostering advancements in personalized health and nutrition guidance.

## Figures and Tables

**Table 1:** The evidence of contribution 1.

Detail	Evidence
Propose recipe nutritional components.	In Section 4.2, a detailed account is presented.
Comprehensive nutritional information is provided to users.	Seven nutritional components of each recipe are extracted from the dataset.
Enhanced dietary management for users.	Users can select recommended recipes based on their current physical condition and the nutritional components of the recipes.

**Table 2:** The evidence of contribution 2.

Detail	Evidence
Propose user nutrition-related food preferences.	In Section 4.3, a detailed account is presented.
Tailored recipe recommendations.	Recipes can be recommended based on users’ nutritional preferences.
Aggregated user nutritional preferences.	After extracting the nutritional components of the recipes, they are combined with the user.

**Table 3:** The evidence of contribution 3.

Detail	Evidence
Propose a novel nutrition-related knowledge graph food recommendation method based on a GCN.	In Section 4, we provide an overview of the entire methodology.
Capturing richer and more comprehensive information.	By extracting nutritional components from recipes, calculating the similarity between recipes, and incorporating user nutritional preferences, the methodology learns more useful information.
Improved efficiency and accuracy of recommendations.	In Section 5.4, a detailed comparison with other methods shows significant improvements in ACC and precision metrics.

**Table 4:** The evidence of contribution 4.

Detail	Evidence
Superior performance compared to six baseline methods.	In Section 5.4, our method shows improvements over the baseline on all five evaluation metrics.
Demonstrated potential and effectiveness.	In Section 6, we illustrate the superiority of our method through two examples.

**Table 5:** Comparison of machine learning and neural network methods and their application domains in related work.

Related Work	Machine Learning	Neural Network	Application Domain
He et al. [20]	✓	Deep neural network	E-commerce
Wang et al. [21]	✓	Graph neural network	E-commerce
Zhang et al. [22]	✓	Graph attention network	E-commerce
Yin et al. [23]	✓	Graph convolutional network Graph attention network	E-commerce
Ge et al. [24]	✓	×	Food
Chen et al. [25]	✓	Graph convolutional network	Food
Gao et al. [26]	✓	Graph convolutional network	Food
Song et al. [27]	✓	Graph neural network	Food
Rostami et al. [28]	✓	×	Food
Our	✓	Graph convolutional network Graph attention network	Food

**Table 6:** Comparison of nutritional components, user preferences, and heterogeneous graph usage in related work.

Related Work	Nutritional Components	User Preferences	Heterogeneous Graph
He et al. [20]	×	×	×
Wang et al. [21]	×	×	×
Zhang et al. [22]	×	Point of interest	Heterogeneous directed graph
Yin et al. [23]	×	Geometric relationships in the space	×
Ge et al. [24]	×	User food ingredient preferences	×
Chen et al. [25]	Sodium, fat, sugar, saturated fat	User dietary preferences	×
Gao et al. [26]	×	×	×
Song et al. [27]	Calories	User calorie preference	Heterogeneous directed graph
Rostami et al. [28]	×	×	×
Our	Calories, total fat, saturated fat, sodium, protein, sugar, carbohydrates	User nutrition-related food preferences	Heterogeneous undirected graph

**Table 7:** List of essential notations.

Essential Notation	Description
$U=\{u_{1},u_{2},\ldots ,u_{M}\}$	Set of users
$R=\{r_{1},r_{2},\ldots ,r_{N}\}$	Set of recipes
Θ	User–recipe interaction matrix
G	Heterogeneous undirected graph data
ϑ	Recipe nutritional components
T	User recipe ratings
ϰ	Nutrition-related food similarity graph
P	User nutrition-related food preferences
$\tilde{u}$	User embedding
$\tilde{r}$	Recipe embedding
Z	Recipe information table

**Table 8:** Drawbacks of related work.

Related Work	Drawbacks
Ge et al. [24]	Nutritional components not considered; nutritional balance is disregarded solely based on user preferences.
Chen et al. [25]	Nutritional components included are fewer (four types); high costs for data acquisition and maintenance.
Gao et al. [26]	Nutritional components not considered; only explored internal knowledge associations among ingredients, recipes, and users, neglecting external knowledge such as ingredient–disease relationships.
Song et al. [27]	Nutritional components included are fewer (one type); considering only food calories could lead to unhealthy recommendations.
Rostami et al. [28]	Nutritional components not considered; comprehensive use of graph clustering, deep learning, and multiple information sources may lead to high system complexity, impacting scalability and maintenance costs.

**Table 9:** Different methods had varying performance metrics on datasets of 10% and 50% proportions.

Model	Food									
	10%					50%				
	AUC	ACC	Precision	Recall	F1	AUC	ACC	Precision	Recall	F1
FeaStNet	0.8849	0.8072	0.8490	**0.7473**	**0.7949**	0.7998	0.7267	0.7500	**0.6800**	0.7133
DeeperGCN	0.8285	0.7326	0.8480	0.5667	0.6794	0.8243	0.7317	0.8013	0.6162	0.6967
GraphSAGE	0.8644	0.7827	0.8478	0.6890	0.7602	0.8391	0.7343	0.8142	0.6073	0.6957
GAT	0.8557	0.7959	0.8534	0.7147	0.7779	0.8396	0.7120	0.7962	0.5697	0.6642
UniMP	0.8630	0.7851	0.8443	0.6992	0.7649	0.8618	0.7600	0.8258	0.6591	0.7331
GATV2	0.8586	0.8037	0.8535	0.7332	0.7888	0.8551	0.7211	0.8061	0.5824	0.6762
Ours	**0.8950**	**0.8145**	**0.9274**	0.6824	0.7863	**0.8723**	**0.7789**	**0.8490**	0.6784	**0.7542**

**Table 10:** Differentmethods had varying performancemetrics on datasets of 80% and 100% proportions.

Model	Food									
	80%					100%				
	AUC	ACC	Precision	Recall	F1	AUC	ACC	Precision	Recall	F1
FeaStNet	0.8231	0.7198	0.7861	0.6041	0.6832	0.8045	0.7079	0.7211	0.6780	0.6989
DeeperGCN	0.8259	0.7436	0.7909	0.6624	0.7210	0.8247	0.7521	0.8052	0.6651	0.7285
GraphSAGE	0.8594	0.7658	0.8170	0.6851	0.7452	0.8613	0.7712	0.8301	0.6820	0.7488
GAT	0.8575	0.7477	0.8088	0.6487	0.7200	0.8685	0.7621	0.8255	0.6648	0.7365
UniMP	0.8713	0.7750	0.8378	0.6819	0.7519	0.8686	0.7796	0.8244	0.7106	0.7633
GATV2	0.8624	0.7519	0.7976	0.6751	0.7312	0.8718	0.7687	0.8057	0.7082	0.7538
Ours	**0.8916**	**0.8131**	**0.8487**	**0.7621**	**0.8031**	**0.8967**	**0.8169**	**0.8427**	**0.7798**	**0.8098**

**Table 11:** The compatibility between user nutrition-related food preferences and nutritional components of recipes.

User Id	User	User Nutrition-Related Food Preferences	Recipe Id	Nutritional Components of Recipes	ρ
480	Emily	453 39 59 19 39 45 11	7712	95 12 0 1 0 15 1	0.9833
775	Jack	555 66 55 17 25 50 8	162	259 24 51 3 14 9 8	0.9908
1484	Lily	319 31 31 14 21 43 6	7038	192 12 7 9 20 15 5	0.9947
346	Tom	509 36 109 20 29 53 19	741	195 9 56 10 7 17 10	0.9954
623	Sarah	407 23 47 37 40 20 17	4070	151 8 21 13 6 3 7	0.9972
3414	David	448 23 219 17 10 37 24	9402	461 38 5 90 96 72 3	0.8101

## Data Availability

The original contributions presented in the study are included in the article, further inquiries can be directed to the corresponding author.
